# Targeting Transcriptional Regulators of CD8+ T Cell Dysfunction to Boost Anti-Tumor Immunity

**DOI:** 10.3390/vaccines3030771

**Published:** 2015-09-17

**Authors:** Katherine A. Waugh, Sonia M. Leach, Jill E. Slansky

**Affiliations:** 1 University of Colorado School of Medicine, 12800 East 19th Avenue, Mail Stop 8333, Aurora, CO 80045, USA; E-Mail: Katherine.Waugh@UCDenver.edu; 2 Center for Genes, Environment and Health, National Jewish Health, Denver, CO 80206, USA; E-Mail: LeachS@NJHeath.org

**Keywords:** tumor-infiltrating T cells, TIL, transcriptional regulation, T cell dysfunction, T cell hypofunction, anergy, tolerance, exhaustion, NF-κB, NFAT, PD-1, pathway analysis, transcriptome analysis, cancer immunotherapy

## Abstract

Transcription is a dynamic process influenced by the cellular environment: healthy, transformed, and otherwise. Genome-wide mRNA expression profiles reflect the collective impact of pathways modulating cell function under different conditions. In this review we focus on the transcriptional pathways that control tumor infiltrating CD8+ T cell (TIL) function. Simultaneous restraint of overlapping inhibitory pathways may confer TIL resistance to multiple mechanisms of suppression traditionally referred to as exhaustion, tolerance, or anergy. Although decades of work have laid a solid foundation of altered transcriptional networks underlying various subsets of hypofunctional or “dysfunctional” CD8+ T cells, an understanding of the relevance in TIL has just begun. With recent technological advances, it is now feasible to further elucidate and utilize these pathways in immunotherapy platforms that seek to increase TIL function.

## 1. Brief History of Cancer Immunotherapy

In the late 19th century, New York surgeon William Coley documented spontaneous elimination of sarcomas in patients suffering from erysipelas, an acute streptococcal infection [1,2]. Coley then utilized this observation to develop a radical cancer treatment—he injected live *Streptococcus pyogenes* directly into cancer patients. Some patients, especially those suffering from spindle cell sarcomas, were permanently cured of tumors that had previously been deemed inoperable and “entirely hopeless”. Tumor regression occurred in some patients after treatment with live bacteria regardless of whether or not they showed symptoms of erysipelas, but injection of heat-killed bacteria had a reduced effect on tumor regression. Therefore, to increase virulence but reduce patient discomfort from erysipelas, Coley worked with others to optimize production and delivery of a therapeutic anti-cancer vaccine containing mixed toxins, or Coley’s Toxins, from *S. pyogenes* and *Serratia marcescens*. Vaccination elicited an inflammation storm and permanently cured up to 40% of his patients. Despite potential pitfalls underlying Coley’s trials and a lack of mechanistic understanding, these studies were the first to demonstrate that the immune system can be activated to treat cancer.

Around the same time, German physician Paul Ehrlich was also applying bacteriological methods to cancer research. He reasoned that since a chemical dye selectively stains a microorganism or tissue, a similarly selective agent could be used to specifically target and kill microorganisms or transformed cells and leave surrounding tissues untouched like a “magic bullet” [3]. The magic bullet concept has recently been reintroduced in tumor immunology with the development of monoclonal antibodies as treatments that provide specific delivery of toxic agents to tumors as well as blockade of receptors that inhibit function of immune cells against tumors.

Even with Coley’s promising clinical trials, Ehrlich’s desire to activate the immune system to specifically kill cancer cells was not extensively pursued for about 50 years [3]. With the development of inbred mouse strains, collective work by E.J. Foley, Georg Klein, Lloyd Old, and Edward Boyse determined tumors were immunologically distinct from normal tissues [4,5,6,7,8]. These and other relevant studies prompted Frank Macfarlane Burnet and Lewis Thomas to formally introduce the cancer immunosurveillance hypothesis [8,9,10]. They hypothesized that mutations flag continuously-arising nascent-transformed cells for specific destruction by lymphocytes. However, the studies that followed were unknowingly ill-equipped to test the hypothesis. For instance, nude mice acquired tumors at similar rates as wild type mice [11]. These mice were later determined to be immunocompromised rather than immunedeficient through spontaneous deletion of the Foxn1 gene that facilitates T cell development. The rate of tumor development in nude mice in this study and other seeming contradictions led many to reject the cancer immunosurveillance hypothesis for decades [8].

By the early 21st century, Robert Schreiber and colleagues updated the cancer immunosurveillance hypothesis into a more comprehensive “cancer immunoediting” model [8]. This model was advanced by the deeper molecular understanding of the immune response thanks to the advent of new technologies such as gene targeting, transgenic methods, and the production of monoclonal antibodies. As the field of tumor immunology has grown, we have learned that diverse interactions of the immune system with neoplastic tissues shape an evolving tumor environment and can also facilitate tumor growth.

Starting in the late 1970s, Belgian scientist Thierry Boon and colleagues significantly advanced the field by showing cancers were indeed immunogenic and recognized by T cells. By dissecting the anti-tumor immune response in both mice and human samples, they were the first to clone tumor antigens and T cells that recognized tumor antigens. Numerous categories of tumor antigens were identified including those that were associated with tumors, but were also identified on normal tissues (tumor-associated antigens) and those derived from mutations (tumor-specific rejection antigens) [12,13].

In the last decade, we have built heavily on the foundation of early cancer immunoediting studies. For some cancers, immune infiltration data from tumors, or “immunoscores”, are better predictors of cancer patient prognosis than traditional methods that distinguish different stages of disease [14,15,16]. In particular, there is compelling evidence that associates localized accumulation of functional tumor-infiltrating CD8+ T cells (TIL) with increased survival of cancer patients [8,17,18,19]. The most desirable collective phenotype of multifunctional TIL includes production of cytokines such as interferon gamma (IFNγ), toxicity toward target cells, and proliferative capacity [8]. However, tumors evolve to suppress, evade, and even manipulate the immune system to promote tumor growth [8,20]. Ultimately, TIL usually have a hypofunctional phenotype incapable of tumor clearance [21].

## 2. Rationale to Pursue Transcriptional Regulation of TIL Dysfunction

The promise of eliciting functional anti-tumor immune response for cancer treatment has been developing for decades to result in a recent explosion of clinical triumphs [22]. Current therapies successfully utilized in the clinic to boost anti-tumor immunity include diverse methods of active and passive immunization [23,24]. Efforts to increase TIL function generally seek to expand the number of functional anti-tumor T cells, or to reduce immunoinhibitory stimuli present in the patient’s tumor environment [23]. However, immunotherapies that target a single pathway are often ineffective against established solid tumors [25,26,27].

Co-therapies used to target multiple pathways inhibiting TIL function are being translated from mouse models to the clinic [25,26]. For instance, dual blockade of T cell inhibitory receptors programmed death-1 (PD-1 or CD279) and cytotoxic T-lymphocyte-associated protein 4 (CTLA-4 or CD152) has greatly increased progression-free survival and overall response rates of an encouraging fraction of patients bearing previously incurable solid tumors [24,25,26,27,28]. Approximately 50% of patients with advanced melanoma experienced tumor regression, and ongoing clinical trials, show promise in a variety of malignancies. Although anti-tumor responses may not clear all tumors, other promising combinatorial immunotherapies are in trials [23]. Current research is largely focused on predicting which cancer patients will benefit from specific immunotherapies [24,29].

Tumor-orchestrated mechanisms of immunosuppression that effectively restrict TIL function include chronic suboptimal antigen stimulation, soluble and cell-bound factors expressed by the tumor cells or stroma, and an overall toxic environment with low levels of nutrients and oxygen [30,31]. The sheer number of inhibitory mechanisms capable of restricting TIL function makes efficient identification of effective treatment combinations for individual tumors a daunting task. Furthermore, adding additional therapies to those already combined increases the cost and risk of adverse toxicities [25,32].

**Figure 1:**
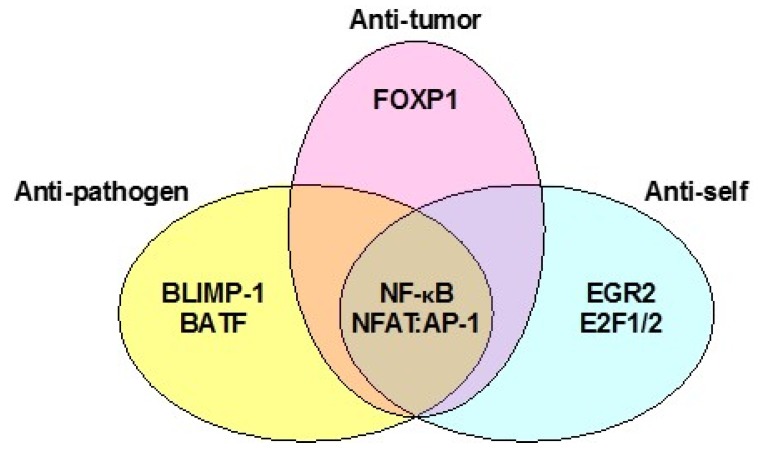
Overlap of key transcriptional networks underlying hypofunction of anti-tumor, exhaustion of anti-pathogen, and tolerance or anergy of anti-self CD8+ T cells.

Many inhibitory pathways eventually converge downstream to restrict the same functions in T cells. Although the relay between many inhibitory signals and decreased function of TIL remains a black box, this body of literature is growing fast, as discussed below. CD8+ T cell perception of a unique array of extracellular signals generated by assaulting pathogens or malignancies are collectively reflected in genome-wide mRNA expression profiles, or “transcriptomes”. Recent studies have shown that various states of T cell dysfunction relevant to TIL bear distinct, yet overlapping, transcriptomes that relay phenotypic and functional readouts of hypofunction (Figure 1) [33,34,35,36,37]. As reflected by the focus of current literature, transcriptional regulators of T cell hypofunction could therefore be of great value to the development and prediction of successful immunotherapies.

## 3. Transcriptional Regulators of Anergy and Tolerance in TIL

**Figure 2:**
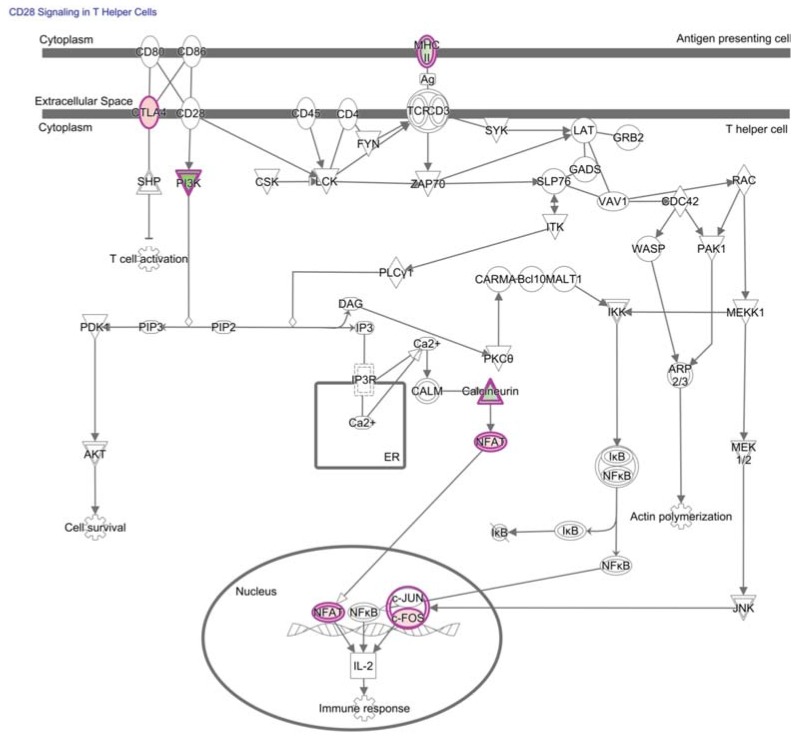
TAA-specific CD8+ T cells often lack co-stimulation for an immune response against tumors. CD28 signaling was reflected in a top canonical pathway predicted by genome-wide mRNA expression profiling to trend towards less activation in TAA-specific CD8+ T cells from the tumor compared to those in circulation (*p*-value of overlap = 3.2E-4, right-tailed Fisher Exact Test, z-score = −0.816). Many signaling pathways overlap between T cell subsets, so it is not surprising that a CD4+ T cell signaling pathway was associated with the gene expression of CD8+ T cells used to generate the figure above. Differential gene expression corresponding to molecules enriched in this pathway are outlined in purple with red fill representing overexpression and green fill representing decreased expression in T cells from the tumor relative to the periphery, and color intensity corresponds to the extent of expression difference. CD28 was predicted to be a key upstream regulator that is less active in TAA-specific CD8+ T cells from the tumor (*p*-value of overlap = 1.3E-5, right-tailed Fisher exact test, activation z-score = −0.696). CTLA-4 was also predicted to be an upstream regulator of differential gene expression (*p*-value of overlap = 9.7E-5, right-tailed Fisher exact test). Differentially-expressed genes and corresponding fold-changes have been previously published and were reanalyzed through the use of QIAGEN’s Ingenuity^®^ Pathway Analysis (IPA^®^, QIAGEN, Redwood City, CA, USA, www.qiagen.com/ingenuity) [35]. Both direct and indirect relationships were assessed in the Ingenuity knowledge base reference set with a confidence threshold of previous experimental observation in T cells. Note: The connecting arrow between SHP and T cell activation was altered to a line to better reflect inhibition downstream of CTLA-4.

Many tumor antigens recognized by T cells have been identified including tumor-specific antigens (TSAs) generated by mutations and tumor-associated antigens (TAAs) generated by overexpressed non-mutated proteins [38]. T cell antigen receptor (TCR) affinity influences tolerogenic mechanisms and, typically, high-affinity interactions with self-antigens delete or tolerize T cells [39,40]. Upon activation, the remaining self-reactive T cells may become functionally competent to mediate autoimmune disease and tumor destruction [40,41]. However, the T cell interaction with TAAs is often of too low affinity or lacks the necessary immunostimulatory co-signals for T cell expansion and elimination of tumor cells [21]. Figure 2 illustrates a canonical pathway in TAA-specific TIL involving CTLA-4 competition with co-signal delivered by the surface receptor CD28 during TCR stimulation. 

**Figure 3:**
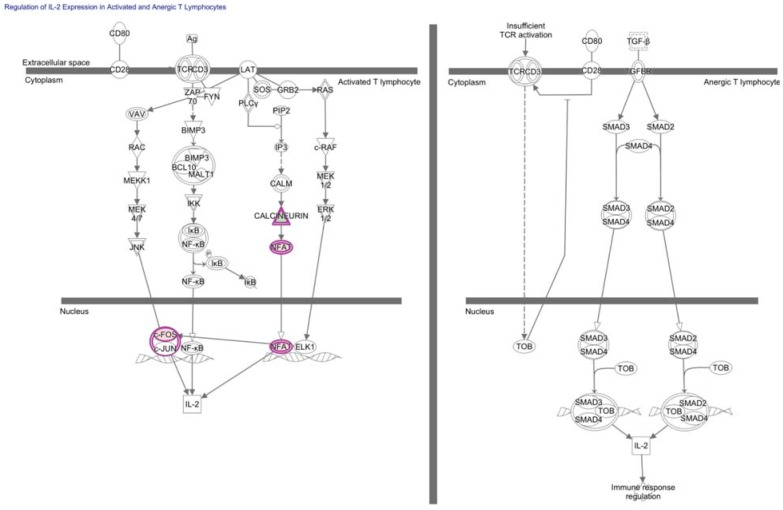
Activated and anergic T cells differentially relay extracellular signals to the IL-2 locus. Regulation of IL-2 Expression in Activated and Anergic T Lymphocytes is a canonical pathway enriched for molecules corresponding to differentially-expressed genes of tumor-specific CD8+ T cells from the tumor relative to the periphery (*p*-value = 4.9E-2, right-tailed Fisher Exact Test). These molecules are outlined in purple with red fill representing overexpression and green fill representing decreased expression in T cells from the tumor relative to the periphery, and color intensity corresponds to the extent of expression difference. Differentially-expressed genes and corresponding fold-changes have been previously published and were reanalyzed through the use of QIAGEN’s Ingenuity^®^ Pathway Analysis (IPA^®^, QIAGEN, Redwood City, www.qiagen.com/ingenuity) [35]. Both direct and indirect relationships were assessed in the Ingenuity knowledge base reference set with a confidence threshold of previous experimental observation in T cells.

Sub-threshold TCR stimulation can maintain TAA-specific T cells in an un-activated or antigen-inexperienced state in which T cells remain “ignorant” of a growing tumor and generally do not control tumor growth [17,42,43]. Upon threshold TCR stimulation, T cells lacking additional immunostimulatory co-signals undergo activation-induced cell death or progress along a transcriptional program to tolerance or anergy. Because of molecular and phenotypic likeness, the historically loaded terms “anergy” and “tolerance” have often been interchanged. Both are generally characterized by lack of proliferation, absence of cytokine production such as IL-2, and inability to kill target cells in response to self-antigen (Figure 3) [37,44]. Distinctions between the overlapping anergic and tolerant T cell states have been recently reviewed [37]. We focus on transcriptional regulators of anergy and tolerance because they are both relevant to heterogeneous anti-tumor T cell responses. Importantly, anergy and tolerance programs can be overcome to generate functional CD8+ T cells and augment anti-tumor immunity. Promising strategies to mobilize TIL function specifically against TAAs include TCR-based gene therapies, chimeric antigen receptors (CARs), and dendritic cell vaccines loaded with antigens [23,45,46,47].

The transcriptome of tolerant CD8+ T cells is distinct from other T cell programs, as shown in a mouse model [36]. Compared to memory and naïve T cells, functionally tolerant CD8+ T cells have increased expression of the early growth response 2 (Egr2) transcription factor and of E2F transcription factors 1 and 2 (E2F1 and E2F2). Overexpression of Egr2 shortly after TCR stimulation inhibits T cell activation, and the critical role of Egr2 in the induction of anergy *in vitro* and *in vivo* is an active area of intensive research [48,49,50,51]. Although E2F1 has been shown to be a cell cycle progressor in many cellular contexts, studies show that in murine CD8+ T cells, E2F1 and E2F2 redundantly restrict cell cycle progression and proliferation following sub-threshold antigen stimulation, and mice are more prone to autoimmunity [52,53,54,55,56,57,58,59,60]. E2F1 also regulates activation induced cell death in T cells through an undefined pathway downstream of the TCR [59,60].

Not surprisingly, tolerant CD8+ T cells also have decreased expression of many effector molecules and of transcription factors known to control T cell function, such as T-box 21 (Tbx21 or T-bet), Eomesodermin (Eomes), GATA-binding protein 3 (Gata3), and signal transducer and activator of transcription 4 (Stat4) [36,37,61,62,63]. Alternative expression of chromatin modifiers and miRNAs, such as microRNA-181a, also accompany T cell commitment to the tolerant state [36,64,65]. This list of transcriptional regulators identified in tolerant CD8+ T cells will be an invaluable resource for functional studies in TIL.

A recent study compared wild type to Egr2-deleted CD4+ T cells under anergizing conditions [33,51]. Zheng *et al.*, merged chromatin immunoprecipitation sequencing with gene expression profiling to generate a list of genes that is directly regulated by Egr2 in anergic CD4+ T cells [51]. This list of 49 genes overlaps with transcripts that are highly expressed by tolerant CD8+ T cells and hypofunctional TIL, such as the negative regulator of T cell function, lymphocyte-activation gene 3 (Lag3) [36,66]. Such analyses may provide a valuable list of novel therapeutic targets in TIL downstream of Egr2 regulation of T cell anergy.

## 4. NF-κB in Hypofunctional Anti-Self and Tumor Infiltrating CD8+ T Cells

Nuclear factor (NF)-κB is a family of structurally related transcription factors, whose function has been heavily studied as a fundamental regulator of immune responses [67]. In T cells, NF-κB regulates a wide range of gene expression including those underlying development, activation, differentiation, and function. After TCR engagement, activation of NF-κB is critical for cytokine production and T cell survival. NF-κB signaling can promote or prevent chronic inflammation as well as autoimmunity, and is, therefore, tightly controlled [67,68]. In resting T cells, the NF-κB factors are normally associated with inhibitory proteins and sequestered in the cytoplasm. Various immune stimuli lead to degradation of these inhibitory proteins, generation of mature NF-κB complexes, translocation to the nucleus, and transactivation of many genes by NF-κB hetero- or homodimers through binding to a κB enhancer. Multiple signaling pathways throughout an immune response influence NF-κB-mediated gene transcription. For example, alternative functional outcomes result from transient activation of NF-κB during an acute immune response and chronic activation of NF-κB during persistent infections or cancers.

Although anergy has been most heavily studied in CD4+ T cells, anergic CD8+ T cells have defective NF-κB-mediated gene transcription; the cytokine IL-2 is not transcribed due to decreased modifications of the NF-κB subunit RelA/p65 [69]. Hypofunctional T cells from the periphery of tumor-bearing mice express less RelA/p65-p50 and alternative forms of p50 bound to DNA that are restored to normal upon successful immunotherapy [70]. In patients, early studies using delayed-type hypersensitivity (DTH) as a read-out showed that responsiveness to a TAA-based vaccine against solid tumors also positively correlates with RelA/p65 levels in T cells [71]. Such studies originally defined T cell anergy as the absence of DTH to recall antigens in cancer patients [72,73]. These findings were among the first to suggest that transcriptional regulators of anti-tumor T cell function should be monitored during clinical trials to indicate responses to cancer treatment.

Molecular studies regarding consequences of NF-κB signaling in T cell function have since proven complex and are outside the scope of this review [67]. However, TCR signaling induces activation of NF-κB, and many suppressive factors produced by tumors can inhibit TCR-induced NF-κB activation. So, although NF-κB activity is blunted in hypofunctional T cells that often have reduced TCR components in tumor-bearing hosts, whether reduced NF-κB signaling in T cells is a cause or an effect of the inability to control tumor growth was unknown until the following study [74]. Barnes *et al.*, utilized mice that have impaired NF-κB signaling in T cells, to determine that NF-κB signaling is required for tumor elimination [74]. Although many questions remain regarding specific roles of NF-κB in TIL, this study strongly suggests that NF-κB may be worth more in TIL than just a read-out of treatment responses. Implications of this study will be further explored below as there are also negative allegations against enhanced NF-κB signaling downstream of an inhibitory receptor overexpressed on T cells during chronic TCR stimulation.

## 5. TIL and JAK/STAT Relay of Extracellular Signals for T Cell Programming

The Janus kinase (JAK)/STAT and phosphatidylinositol 3-kinase/protein kinase B (PI3K/PKB or AKT) pathways relay a vast array of extrinsic and intrinsic stimuli to alter transcription of T cells [75]. Upon receptor-ligand binding, associated JAKs become activated through trans-phosphorylation to phosphorylate their major substrates, STATs. Phosphorylation of these cytoplasmic transcription factors facilitates dimerization, nuclear translocation, and activation or repression of target gene transcription. Cytokines are key initiators of JAK/STAT pathways that can lead to T cell subset polarization; however T cell programs remain incredibly plastic as reflected by shifting gene expression profiles and functional capacities [76]. 

**Figure 4:**
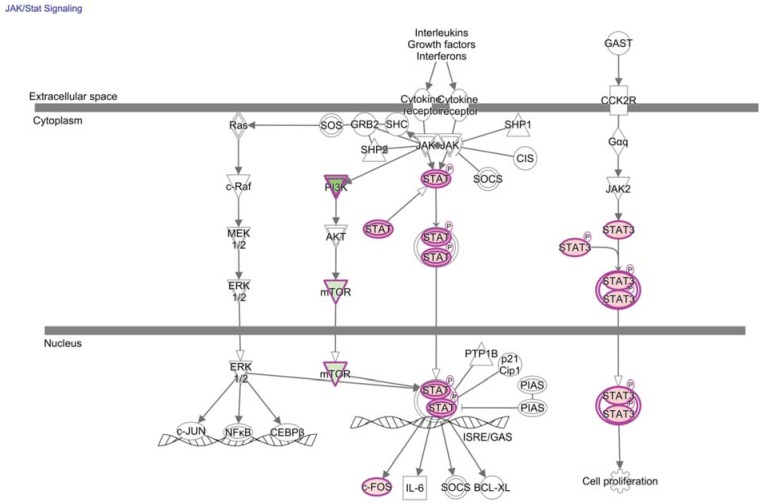
JAK/STAT signaling directly relays extracellular signals to transcription in T cells. JAK/Stat signaling is a canonical pathway enriched for molecules corresponding to differentially expressed genes of tumor-specific CD8+ T cells from the tumor relative to the periphery (*p*-value = 3.5E-2, right-tailed Fisher Exact Test). These molecules are outlined in purple with red fill representing overexpression and green fill representing decreased expression in T cells from the tumor relative to the periphery, and color intensity corresponds to the extent of expression difference. Differentially-expressed genes and corresponding fold-changes have been previously published and were reanalyzed through the use of QIAGEN’s Ingenuity^®^ Pathway Analysis (IPA^®^, QIAGEN Redwood City, www.qiagen.com/ingenuity) [35]. Both direct and indirect relationships were assessed in the Ingenuity knowledge base reference set with a confidence threshold of previous experimental observation in T cells.

Transcriptional consequences of JAK/STAT signaling pathways in the tumor environment have largely been in the cellular context of other immune or tumor cells rather than TIL [77]. There is, nevertheless, a prevailing perception that STAT3 signaling in the tumor environment is detrimental to anti-tumor immune function and represents a promising target to refocus tumor-promoting inflammation [77]. However, Triplett *et al.*, showed increased levels of phosphorylated STAT3 in TIL after successful treatment of large established tumors with a co-therapy that boosts effector T cell function with an agonist antibody directed against tumor necrosis factor receptor superfamily, member 4 (TNFRSR, CD134, or OX40) and a small molecule inhibitor of the transforming growth factor beta (TGF-β) receptor [78]. Genetic deletion of STAT3 in OX40-expressing cells also significantly reduced treatment efficacy [78]. Triplett’s study favors a model in which STAT3 signaling is not detrimental to TIL function and suggests that STAT3 may have opposing roles depending on the environment of TCR engagement as suggested in Figure 4. STAT3 may, therefore, represent a promising target to boost anti-tumor function of TIL, but must be thoroughly investigated in proposed therapeutic platforms.

## 6. Transcriptional Regulators Underlying PD-1 Signaling and Expression

Recent studies focus on transcriptional programs downstream of inhibitory receptors that are highly expressed by TIL; for example, PD-1 inhibits cell cycle progression of T cells following TCR stimulation [79]. *In vitro*, PD-1 stimulation initiates transcriptional repression of S-phase kinase-associated protein 2 (Skp2), the substrate recognition component of the Skp, Cullin, F-box (SCF) complex that catalyzes ubiquitination of proteins for protesomal degradation [80,81]. Subsequent accumulation of p27^Kip1^ protein impairs cyclin-dependent kinase 2 (CDK2), the downstream functions of retinoblastoma (Rb), and the transcription factor mothers against decapentaplegic homolog 3 (SMAD3). Subsequent reduced transcription of E2F target genes and enhanced SMAD3 activity, respectively, lead to alterations in cell cycle machinery and, ultimately, to cell cycle arrest in the G1 phase. Although decreased transcription of Skp2 ultimately mediates PD-1 restriction of cell cycle progression in T cells, the transcriptional regulators responsible are unknown, but are mediated by inhibition of PI3K/AKT as well as RAS/MEK/ERK pathways and incubation with IL-2 restores phosphorylation of MEK/ERK but not AKT proteins.

PD-1 inhibition of cell cycle progression fits well into the context of studies *in vivo* and T cell anergy in particular. Increased levels of p27^Kip1^ positively correlate with cell cycle arrest of human and mouse CD4+ T cells anergized *in vitro* and murine CD4+ T cells anergized *in vivo* [82,83,84,85,86]. Productive TCR signaling paired with immunostimulatory CD28 co-stimulation is also necessary for downregulation of p27^Kip1^ through activation of PI3K/AKT pathways in primary human T cells [87]. As stated above, when such immunostimulatory co-signals are absent during TCR stimulation, T cells become anergic or tolerant to restrict autoimmunity. The congruence between anergic T cells and PD-1 restriction of cell cycle progression through p27^Kip1^ is interesting because, although PD-1 is thought to limit autoimmunity, it is not often linked in current literature with anergy [79,88,89].

Other transcriptional regulators in hypofunctional CD8+ T cells that are upstream of PD-1 expression or downstream of PD-1 signaling are also under heavy investigation. For example, PD-1 signaling alters expression of transcription factors STAT1, interferon regulatory factor 9 (IRF9), and basic leucine zipper transcription factor, ATF-like (BATF) [79,90]. In human T cells, knockdown of BATF reduced PD-1 inhibition while enforced expression of BATF decreased cytokine production and proliferation [90]. BATF belongs to the activator protein 1 (AP-1) family of transcription factors and interacts with members of the IRF family [91]. Additionally, IRF9 is an understudied IRF family member that interacts with phosphorylated STAT1:STAT2 dimers to facilitate binding to interferon-stimulated response elements [92]. Subsequent transcriptional activation of corresponding genes drive a cell into an antiviral state in which proliferation is restricted. Although little is known in the context of CD8 T cells, future studies may identify cooperation downstream of PD-1 signaling between STAT1, IRF9, and BATF to restrict TIL function.

Conversely, upstream transcriptional regulators that increase or enforce expression of PD-1 include T-bet, PR domain-containing 1 with ZNF domain (PRDM1 or BLIMP-1), Forkhead box protein O1 (FoxO1), nuclear factor of activated T cells (NFATc1), and mechanisms underlying epigenetic control of the locus that encodes PD-1 [79,93,94,95,96,97]. However, much of the interplay between upstream pathways and downstream transcriptional regulators is largely unknown and unexplored in TIL [79].

## 7. NFAT in Hypofunctional Anti-Self and Tumor Infiltrating CD8+ T Cells

The NFAT family of transcription factors has been heavily studied in the induction and maintenance of T cell activation, anergy, and tolerance [44,98,99,100]. In resting T cells, NFAT1 (NFATc2), NFAT2 (NFATc1), and NFAT4 (NFATc3) are heavily phosphorylated and reside in the cytosol [44]. Calcium flux in response to TCR stimulation induces dephosphorylation and subsequent nuclear localization of these NFAT family members. In stimulated T cells, NFAT proteins form ternary complexes with members of the AP-1 family, Fos and Jun, to elicit specific gene expression. Various stages of this response influence whether or not NFAT-mediated gene expression will initiate or maintain a functional or non-functional T cell response. These factors include the NFAT protein itself, calcium levels, kinase/phosphatase competition for various NFAT residues, cellular localization, efficiency of NFAT:AP-1 complex formation, and an open or closed chromatin state of target binding sites.

Through NFAT, members of the Ikaros family of transcription factors, Ikaros (IKZF1) and Helios (IKZF2), have been tied to decreased IL-2 transcription in CD4+ T cells [101,102]. NFAT-dependent increase in expression of Ikaros in anergic T cells leads to increased IL-2 promoter occupancy, recruitment of histone deacetylase (HDAC), and stable inhibition of IL-2 gene expression [100,103]. Ikaros has since been shown to restrict autocrine IL-2 production in CD8+ T cells, and Ikaros haplo-insufficiency in CD8+ T cells results in better control of infection and tumor growth [104]. Thus, it will be interesting to determine the various therapeutic platforms in which NFAT can be manipulated to augment anti-tumor immunity.

## 8. Transcriptional Regulators of Exhaustion in TIL

Exhaustion represents a heterogeneous population of hyporesponsive T cells that are unique to the assaulting pathogen or malignancy [105]. Exhausted T cells are thought to be terminally differentiated and are generally defined by accumulation of inhibitory receptors, such as PD-1 and gradual loss of function [37,106,107,108]. Virally exhausted CD8+ T cells have a transcriptome distinct from naive, effector, memory, or anergic T cells, that includes expression of transcription factors BLIMP-1, BATF, T-bet, and Eomes [34,90,95,105,106,109,110,111,112]. While studies have historically focused on BLIMP-1 control of CD8 T cell exhaustion, recent publications have also elucidated FoxO1 and FoxO3 as key transcriptional regulators that integrate a range of extracellular signals to alter CD8 T cell survival and function during chronic viral infections [27,94,113,114,115,116,117].

Although exhausted CD8+ T cells were initially observed and have been most heavily characterized during chronic viral infections, various states of exhaustion in TIL have been repeatedly documented in both murine and human solid tumor microenvironments through functional and phenotypic characterization [35,37,79,118]. This is not surprising because evidence suggests chronic TCR stimulation is a driving force behind both T cell exhaustion during chronic viral infection and TIL dysfunction during tumor growth [31,119].

BLIMP-1 is a transcriptional repressor that has emerged as a master regulator of terminal differentiation in a variety of cell types including T cells [120]. Some BLIMP-1 expression is critical for cytolytic effector function, efficient clearance of chronic viral infections, and homeostasis of short-lived effector and memory T cell differentiation [95,121,122,123,124]. Without BLIMP-1 transcription in hematopoietic cells, mice develop a lethal multi-organ inflammatory disease associated with an accumulation of T cells [122]. High expression of BLIMP-1 impairs CD8+ T cell function, and reduced levels rescue or prevent CD8+ T cell exhaustion during chronic viral infections [90,95]. This “goldilocks” aspect of BLIMP-1 expression in T cells may be an underlying reason it is understudied in TIL.

Direct and indirect targets of BLIMP-1 that repress T cell function have been elucidated [125,126,127,128]. For example, BLIMP-1 represses the Ifng locus directly and indirectly through T-bet repression [125]. Additionally, T cell activation initiates an autoregulatory loop in which IL-2 induces transcription of BLIMP-1 to subsequently repress transcription of Fos. BCL6 directly represses transcription of BLIMP-1; however, under exhaustive conditions high levels of BLIMP-1 repress BCL6 to hinder T cell survival, proliferation, and memory differentiation [125,129]. Finally, BLIMP-1 represses DNA-binding 3 (ID3) to limit T cell persistence [127].

Although little is known, mechanistically, regarding BLIMP-1-mediated transcriptional repression in T cells, downstream epigenetic mechanisms have been dissected in the context of other cell lineages. BLIMP-1 recruits an assortment of chromatin modifying enzymes including methyltransferase G9a, histone demethylase LSD1, histone arginine transferase PRMT5, and histone deacetylases HDAC1/2 [130,131,132,133]. While BLIMP-1 usage of specific chromatin modifiers varies across cell lineages, the overall effect is a closed chromatin conformation resulting in reduced transcription [120]. Although evidence suggests that BLIMP-1 and relevant chromatin modifiers LSD1, DNMT3L, and DNMT3A are all expressed in CD8+ T cells during chronic infection, fundamental transcriptional regulators underlying exhaustion of CD8+ T cells are still largely uninvestigated in TIL [125,133,134,135,136,137,138,139].

## 9. Transcriptome of TAA-Specific CD8+ T Cells Reanalyzed to Identify Transcriptional Networks of TIL Hypofunction

Genome-wide mRNA expression of TAA-specific CD8+ T cells isolated from lymph node metastases (TILN) of vaccinated melanoma patients revealed a transcriptome similar to the exhausted program of murine T cells during chronic infection (Gene set enrichment analysis (GSEA), *p* = 0.01, FDR = 0.09) [34,35]. Conversely, TILN shared some similarities with a compiled list of 29 genes associated with anergy and deletional tolerance, such as CTLA-4, but differed in expression of many genes, such as Egr2 [33,35,140]. However, the authors of this original study did not compare the TILN transcriptome to the more recently available transcriptome of tolerant CD8+ T cells [35,36]. We, therefore, reanalyzed their previously-published TILN data to examine such comparisons. Using GSEA, we found significant similarity between TILN and tolerant CD8+ T cells (data not shown; *p*-value < 0.05 and FDR < 0.05, FDR-corrected *p*-value = 0.04) [35,36,141,142,143,144]. Consequently, less emphasis on distinguishing between exhaustion and tolerance may be advantageous in identification of transcriptional regulators as targets for restoration of function in heterogeneous TIL.

Although BLIMP-1 is cited as a likely regulator of tumor-induced CD8+ T cell exhaustion [35], BLIMP-1 expression levels in TILN was not significantly increased in comparison to tumor-specific CD8+ T cells from the periphery [21]. Nevertheless debate over the role of BLIMP-1 in tumor-specific exhaustion is ongoing as TILN may not be very representative of many hypofunctional TIL from other tumor environments [21,35].

**Figure 5:**
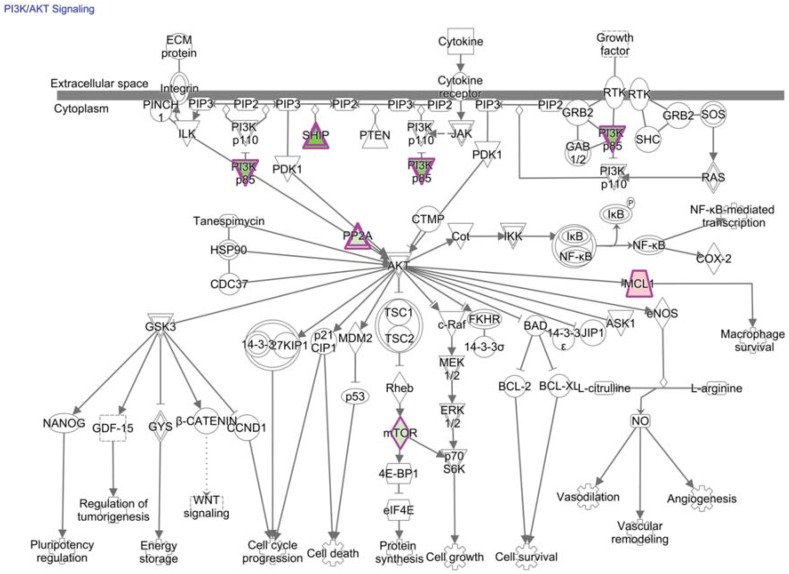
Many signals overlap with PI3K/AKT/mTOR pathways in tumor-specific CD8+ T cells. PI3K/AKT signaling is a canonical pathway enriched for molecules corresponding to differentially expressed genes of tumor-specific CD8+ T cells from the tumor relative to the periphery (*p*-value = 4.1E-2, right-tailed Fisher Exact Test). These molecules are outlined in purple with red fill representing overexpression and green fill representing decreased expression in T cells from the tumor relative to the periphery; color intensity corresponds to the extent of expression difference. mTOR (or FRAP1) was previously published as being down-regulated in TIL and was predicted by our analysis to be an upstream regulator of differential gene expression (fold-change = −3.1, and *p*-value of overlap = 2E-2, right-tailed Fisher Exact Test). Differentially-expressed genes and corresponding fold-changes have been previously published and were reanalyzed through the use of QIAGEN’s Ingenuity^®^ Pathway Analysis (IPA^®^, QIAGEN Redwood City, www.qiagen.com/ingenuity) [35]. Both direct and indirect relationships were assessed in the Ingenuity knowledge base reference set with a confidence threshold of previous experimental observation in T cells.

Few transcriptional regulators met the stringent statistical analysis and three-fold-change cut-off that was used by the original authors to determine the published list of genes that are differentially expressed between TAA-specific CD8+ T cells from the lymph node metastasis and in circulation (Table 1). However, it is not uncommon for transcriptional regulators to be controlled post-translationally, and subtle changes in expression of transcriptional regulators can have global effects. Nevertheless, our additional analysis showed that no transcription factor motifs were convincingly enriched in promoter regions of genes that were published as being statistically up or down-regulated by TILN compared to tumor-specific CD8+ T cells from circulation (*p* < 0.05, FDR < 0.05, Fisher’s exact test, TRANSFAC) [35,145]. To identify transcriptional pathways that may underlie hypofunctional TILN relative to functional tumor-specific CD8+ T cells in circulation, we next applied Ingenuity Pathway Analysis (IPA) to the list of differentially-expressed genes and corresponding fold-changes previously published by the original authors (Figure 2, Figure 3, Figure 4 and Figure 5 and Table 2) [35,145]. As expected, these lists of differentially-expressed genes are predicted to overlap with transcriptional regulators that are associated with anergy, tolerance, and exhaustion.

**Table 1:** Transcriptional regulators are shown among previously published differentially expressed genes in tumor-specific CD8+ T cells in tumor lymph node metastases relative to those in circulation. Differentially-expressed genes and corresponding fold-changes have been previously published and were determined to be transcriptional regulators through the use of QIAGEN’s Ingenuity^®^ Pathway Analysis (IPA^®^, QIAGEN Redwood City, www.qiagen.com/ingenuity) [35].

Gene Probe	Fold change (TILN/PBMC)	Gene Probe	Fold change (TILN/PBMC)
ERF	16.8	ZFP36L1	4.0
HIP2	7.5	ATF4	3.8
CD619445	7.5	ZFP36L1	3.3
AI718865	7.4	IRF4	3.2
ILF2	7.1	E2F1	-3.0
STAT3	7.1	EIF4G3	-4.9
ATF3	6.4	SSBP4	-5.4
BE839843	5.7	SSBP3	-5.5
FOS	5.7	EIF3S9	-5.9
NFAT5	5.3		

**Table 2:** Transcriptional regulators are shown among predicted upstream regulators of all previously published differentially-expressed genes in tumor-specific CD8+ T cells in tumor lymph node metastases relative to those in circulation (*p*-value of overlap <0.05, right-tailed Fisher exact test). Overlap between Table 1 and Table 2, or a direct relationship, is highlighted in green. Differentially-expressed genes and corresponding fold-changes have been previously published and were reanalyzed through the use of QIAGEN’s Ingenuity^®^ Pathway Analysis (IPA^®^, QIAGEN Redwood City, www.qiagen.com/ingenuity) [35]. Both direct and indirect relationships were assessed in the Ingenuity knowledge base reference set with a confidence threshold of previous experimental observation in T cells.

Upstream Regulator	Activation z-score	*p*-value of overlap	Target molecules in dataset
STAT5A	1.342	4.89E-06	CASP8, DUSP5, FASLG, FOS, IFNG, MCL1, S1PR5, TNFRSF25, TNFRSF9, TRAF3
ID3	0	1.05E-04	DUSP1, DUSP4, IFNG, IRF4, NFAT5, PIK3IP1, PIK3R1, TNFRSF25, TRAF3, TRAF5
ID2	0	1.12E-04	DUSP1, DUSP4, IFNG, IRF4, NFAT5, PIK3IP1, PIK3R1, TNFRSF25, TRAF3, TRAF5
FOXP3	-0.555	1.22E-04	CTLA4, DUSP4, ICOS, IFNG, IRF4, RGS1
CYLD		7.31E-04	CTLA4, ICOS, IFNG
STAT5B	1.342	1.07E-03	CASP8, IFNG, MCL1, TNFRSF25, TRAF3
ELF4		1.70E-03	DUSP1, DUSP5
SATB1	-1.741	2.28E-03	DUSP4, PIK3IP1, RGS1, S1PR1, TUBA4A, VTA1
IRF1		2.80E-03	FASLG, IFNG
EGR3		4.16E-03	CBLB, FASLG
JUND		4.16E-03	CTLA4, IFNG
ATF2		4.16E-03	DUSP1, IFNG
NFKB1		5.75E-03	FASLG, IFNG
GATA3		7.12E-03	CTLA4, FOS, ICOS, IFNG
CREB1		7.59E-03	FOS, IFNG
STAT3		7.62E-03	CTLA4, IFNG, IRF4
PRDM1		9.64E-03	FOS, IFNG
HDAC2		1.29E-02	CD27, DCLRE1C, MYO1F
BACH2		1.42E-02	IFNG, IRF4, MCL1
NCOR2		1.71E-02	FOS
IRF2		1.71E-02	FASLG
STAT2		1.71E-02	IFNG
MYBL2		1.71E-02	FASLG
ATF1		1.71E-02	IFNG
NFATC1		1.71E-02	FASLG, IFNG
BCL6		1.99E-02	CTLA4, IFNG, IRF4
HDAC1		1.99E-02	CD27, DCLRE1C, MYO1F
NFATC2		2.97E-02	ICOS, IFNG
NFKBID		3.38E-02	IFNG
TRIM27		3.38E-02	IFNG
CALR		3.38E-02	IFNG
CREBBP		3.85E-02	DGKE, DUSP4, FASLG, IFNG, MYO1F, NR3C1, ST6GAL1
STAT6		4.59E-02	HIPK2, IFNG, IRF4

## 10. NF-κB and NFAT in Exhausted Anti-Pathogen and Anti-Tumor CD8+ T Cells

Virally-exhausted CD8+ T cells and hypofunctional TIL overexpress transmembrane proteins that negatively regulate T cell immune responses, such as PD-1 and TIM-3 [79,146]. PD-1 is a traditional inhibitory receptor in that signaling is initiated through recruitment of tyrosine phosphatases via an inhibitory motif [147]. Conversely, TIM-3 does not bear a known inhibitory motif, but rather binds to the tyrosine kinase Fyn and the regulatory p85 PI3K adaptor [148]. *In vitro*, downstream signaling consequences of TIM-3 in T cells include increased NF-κB- and NFAT:AP-1-mediated transcription as well as cytokine production. As activation of these transcription factors is also a downstream consequence of TCR signaling, TIM-3 may contribute to T cell exhaustion by enhancing TCR-signaling pathways during chronic antigen stimulation [148,149]. *In vivo* data that support this hypothesis show that persistent NF-κB signaling impairs T cell function, survival, and responses to bacterial infection [68]. Although TIM-3 inhibition of TIL function is an active area of investigation, whether TIM-3 restricts T cell function through exacerbating chronic TCR-signaling *in vivo* remains to be elucidated [110,146].

Until recently, additional data regarding NFAT in CD8+ T cell exhaustion were limited and appeared contradictory [21,34,150]. In murine CD8+ T cells, Martinez *et al.*, showed that overexpression of an NFAT1 mutant incapable of binding AP-1 decreases TCR signaling and promotes exhaustion to result in uncontrolled infection and tumor growth [151]. Relative to wild type NFAT1, promoter occupancy of the mutant is also enriched for genes associated with exhaustion. NFAT-mediated transcription lacking AP-1 cooperation therefore drives T cell anergy, tolerance, exhaustion, and TIL dysfunction [98,99,100,151].

## 11. T-Bet and Eomes in Exhausted Anti-Pathogen and Tumor Infiltrating T Cells

As the NF-κB subunit RelA/p65 delineates rescue of anergic T cells in the periphery of cancer patients following immunotherapy, expression levels of T-bet and Eomes delineate reversible CD8+ T cell exhaustion [69,70,71,105]. When exhausted CD8+ T cells are divided into T-bet^hi^ PD-1^int^ and Eomes^hi^ PD-1^hi^, evidence in mice suggests enhanced CD8+ T cell function and viral clearance after blockade of the PD-1:PD-1 ligand pathway is primarily through enhanced proliferation of the T-bet^hi^ PD-1^int^ population [79,105,152]. Comparable phenotypes corresponding to expression levels of T-bet and Eomes in exhausted CD8+ T cells has been documented among patients infected with HIV [153]. While T-bet and Eomes drive T cell subsets, these transcription factors also have context-specific functions, which are relevant but, mechanistically, still largely undefined in the cellular context of TIL [61,109,154,155,156]. Although further studies are necessary to identify the relative abundance of T-bet^hi^ PD-1^int^ to Eomes^hi^ PD-1^hi^ TIL, it may be advantageous to analyze T-bet and Eomes levels in immunoscores to predict cancer patient responses to immunotherapies such as PD-1 blockade [79,156].

## 12. Transcriptional Regulators Underlying TGF-β Inhibition of TIL Function

A T cell’s environment provides distinct signals that direct development, differentiation, and function; TGF-β is an immunosuppressive cytokine enriched in many solid tumor environments [30]. Strategies to reduce tumor inhibition of TIL function by targeting TGF-β include antibodies, antisense oligonucleotides, and small molecules targeting TGF-β receptors [157]. Adoptive transfer of T cells genetically engineered to express a dominant-negative mutant of the TGF-β receptor is also in development. There are many reviews devoted solely to the intricate signaling underlying TGF-β/SMAD signaling, but one additional point in the scope of this review is that TGF-β also restricts self-reactivity of peripheral CD8+ T cells. However, when TGF-β signals are lacking, another cue such as lymphopenia is needed to drive anti-self responses [158,159]. Intracellularly, TGF-β signals are transduced through the SMAD transcription factors. Although data regarding TGF-β/SMAD2/3 regulation of persistent chronic viral infections is conflicting, this signaling pathway is crucial to prevent autoimmunity [160,161,162].

In TIL, SMAD2/3 and NFAT pathways cooperate downstream of TGF-β signaling to enhance anti-tumor immunity by increasing CD103 expression [163]. However, TGF-β is among the suppressive factors in the tumor environment that increases expression of the transcription factor Foxp1 in TIL [164]. To pursue the role of Foxp1 in TIL, Stephen *et al.*, utilized expansion of tumor-specific CD8+ T cells genetically lacking Foxp1 before mechanistic and *in vivo* analyses. In this system, Foxp1 blocks anti-tumor function of murine TIL through transcriptional control of AP-1 complex formation, interacts in the nucleus with SMAD2/3, and is required for inhibition of CD8+ T cell function by TGF-β. Unlike virally-exhausted CD8 T cells, Foxp1 expression is overexpressed to varying degrees by TIL from all queried murine and human tumor environments [109,164].

A recent review of the above study regarding Foxp1 highlights some clinical applications to build on this knowledge of TGF-β signaling, such as engineering anti-tumor T cells without Foxp1 or small molecule disruption of Foxp1-SMAD2/3 or -DNA [157]. In CD8+ T cells, the more heavily studied transcriptional regulator of PD-1 expression, Foxo1, also binds to the same predicted forkhead-DNA binding site as Foxp1 to effect CD8+ T cell differentiation and function, suggesting that manipulation of Foxo1 expression in TIL may be of interest [94,157,165,166]. However, further studies are needed to determine the appropriate therapeutic platforms in which targeting Foxp1 or Foxo1 might be of most use.

## 13. PI3K/AKT/mTOR Signaling and Transcriptional Consequences in TIL

There are many other signals in the tumor environment that are collectively integrated by the residing CD8+ T cell. Many of these signals, such as PD-1, overlap within the PI3K/AKT/mTOR pathway (mechanistic target of rapamycin is abbreviated mTOR) (Figure 5) [79,94,167,168]. Activation of mTOR kinase activity broadly impacts transcription, translation, metabolism, expansion, and differentiation of T cells [167]. mTOR exists in two multi-protein complexes: mTOR complexes 1 and 2 (mTORC1 and mTORC2); upstream stimuli and downstream consequences differ between these complexes [167]. mTORC1 signaling alters gene expression through transcription factors c-MYC and hypoxia-inducible factor-1 alpha subunit (HIF-1α), which are key regulators of gene expression required for metabolic programs in T cells that are likely to affect TIL fitness [167,169].

Levels of stable HIF-1α in the nucleus are increased under hypoxic conditions, like solid tumor environments, in response to growth factors, inflammatory signals, and PI3K/AKT/mTOR activation [170]. To reduce the dangers of systemically activating T cells, clinical trials are taking advantage of targets that have increased expression on T cells located in the hypoxic tumor. For instance, HIF-1α induces high expression of 4-1BB on TIL [171,172]. Conversely, degradation of HIF-1α occurs in normoxia via interactions with the von Hippel–Lindau (VHL) complex, an E3 ubiquitin ligase. Upon deletion of VHL in tumor-specific CD8+ T cells, hypoxia modulates expression of effector molecules through HIF-1α and 2α to enhance function and control of persistent viral infection and tumor growth [173].

## 14. Technological Advances to Utilize Transcriptional Regulators to Increase the Persistence of Functional TIL

Vaccination must induce a persistent immune response, or immunological memory, for protective immunity [119]. The field of research to elicit persistent cellular immunity is outside the scope of this review. However, increased expression of Eomes, B-cell lymphoma 6 (BCL6), TNF receptor-associated factor 6 (TRAF6), and transcription factor 1 (TCF1) promote memory T cells, whereas mTOR, T-bet, and BLIMP-1 promote short-lived T cells [61,123,124,129,174,175,176,177,178,179]. Use of pharmacologic agents to drive T cell differentiation toward memory *in vivo*, such as rapamycin inhibition of mTOR, has risks of broad off-target effects. [167,174,175].

Advances in oligonucleotide aptamer technology have recently produced an exciting increase in target cell specificity [174]. Aptamers are high-affinity, single-stranded nucleic acid ligands with specificity and avidity comparable to antibodies [180]. mTORC1 activity is down-regulated and CD8+ T cells are activated following immunization with siRNA against mTORC1 conjugated to an aptamer targeting 4-1BB, a co-stimulatory molecule expressed after TCR stimulation [174]. This vaccine enhanced memory T cell differentiation and protection from tumor challenge, even though 4-1BB and mTORC1 are also expressed by a regulatory T cell subset that promotes tumor growth [174,181]. Conversely, the systemic administration of a pharmacologic inhibitor of mTORC1 was not as effective at augmenting tumor rejection [174]. Aptamer delivery of RNAi, therefore, offers a reasonable approach to manipulate intracellular pathways in the clinic [174,180,182,183,184,185].

Another strategy to limit systematic effects of agents that alter cellular differentiation is removing T cells from patients to pharmacologically or genetically manipulate intracellular pathways, then adoptively transferring these cells into the cancer patient [186]. Because success of such treatments largely depends on long-term persistence of transferred T cells, generating a large number of anti-tumor T cells that have enhanced self-renewal capacity and multipotency is an area of intense interest. Focus includes differential expression of transcription factors in a memory T cell subset with stem cell qualities such as FOXP1, inhibitor of DNA-binding 3 (ID3), multiple members of the Kruppel-like factor (KLF) family, and the WNT-β-catenin signaling transducers T cell factor 7 (TCF7) and lymphoid enhancer-binding factor 1 (LEF1) [179,186,187,188,189,190,191,192].

Evidence suggests a subset of memory CD8+ T cells with stem cell-like qualities is a precursor that can be differentiated into other T cell subsets [187]. Culture techniques that limit differentiation of anti-tumor T cells away from a more pluripotent memory subset during expansion before adoptive transfer is an area of active research including pharmacologic manipulation of developmental pathways, such as mTOR and AKT inhibitors [193]. However, balancing T cell proliferation and differentiation is difficult during expansion of persistent anti-tumor T cells. Focus may, therefore, shift to reprogramming T cells or differentiating stem cells into functional antitumor T cells. For instance, ectopic expression of transcription factors such as OCT4, SOX2, KLF4, and c-MYC may de-differentiate T cells into induced pluripotent stem cells before reprogramming into a less differentiated T cell that bears the same tumor-specific TCR [186].

## 15. Technological Advances to Identify Novel Transcriptional Regulators of TIL

Highly innovative pipelines have been developed to systematically identify intracellular targets that enhance TIL function *in vivo*. For example, transducing tumor-specific CD8+ T cells with pooled lentiviral shRNA libraries before adoptive transfer into tumor-bearing mice determined that protein phosphatase 2, regulatory B subunit (Ppp2r2d) is a novel target in TIL to enhance immune function [194]. In this system, shRNAs that competitively increased the number of TIL were identified by next generation sequencing and novel hits were pursued functionally. Although the discovery method was more robust in CD8+ T cells specific for the surrogate TSA, OVA, there was also promise in CD8+ T cells specific for a TAA. Many variables between these two T cell populations may have affected expansion, such as higher TCR affinity for a TSA. However, shRNA pools may also have been more relevant to the TSA-specific T cells because the two screens were generated using a broad library targeting kinases/phosphatases or a custom library targeting transcripts associated with CD4+ T cell anergy and CD8+ T cell exhaustion against a non-self viral antigen. Therefore, more robust responses may be obtained in TAA-specific CD8+ T cells if an shRNA library is generated to target transcripts associated with tolerant CD8+ T cells. Knockdown technologies may be especially useful to decrease “goldilocks” transcription factors for which expression is necessary for functional CD8+ T cell responses, but are hypothesized to inhibit TIL function when overexpressed. *In vivo* high-throughput discovery methods offer pipelines to expand our limited knowledge underlying transcriptional regulators of TIL dysfunction and to identify therapeutically relevant targets *in vivo*.

Other genome editing technologies, such as CRISPR-Cas9 sgRNA, could also be used to investigate transcriptional regulators of TIL dysfunction [195]. For instance, the above pipeline could be modified to utilize a custom Edit-R Lentiviral sgRNA Pooled Screening Library (GE Healthcare) to eliminate transcription factors that are associated with decreased CD8+ T cell function. Additionally, the CRISPR-Cas9 system could be utilized to investigate the many transcription factors that are regulated post-translationally, as mutating a single residue, such as an Arginine on E2F1, can drive a cell down widely different paths [196]. Although the small number of anti-tumor T cells is limiting for many of the analyses proposed above, it is now plausible to utilize single cell methods to molecularly investigate a small number of TIL [197]. Such methods could be used to elucidate the intricate transcriptional regulation of heterogeneous TIL dysfunction for therapeutic applications.

## 16. Discussion and Conclusions

T cell programming is a dynamic process intimately linked to an array of environmental signals that lead to differential expression of lineage-specific transcription factors. At any given time in a patient, TAA and TSA-specific CD8+ T cells circulating through the blood, lymph nodes, and tumor tissue are a heterogeneous population at various states of activation and differentiation in response to an evolving malignancy and milieu. Transcriptional control of TIL hypofunction overlaps with programs controlling exhaustion, anergy, and tolerance. These molecular programs are plastic, and external cues temporarily restore T cell function. However, whether these subsets can be reprogrammed into functional TIL that persistently augment anti-tumor immunity in patients remains unanswered.

Co-therapies that target multiple pathways inhibiting TIL function are being translated from mouse models to clinical successes, and efforts are now focused on predicting effective immunotherapy combinations for individual patients [25,26]. We have known for decades that there is a positive correlation between activation of the NF-κB subunit RelA/p65 in T cells circulating in cancer patients and response to immunotherapy. Manipulating transcriptional pathways underlying various states of CD8+ T cell hypofunction, such as NF-κB or NFAT, has since been utilized to significantly augment tumor clearance in mice. Although striking, these findings are not surprising because many inhibitory pathways eventually converge downstream to alter transcription of molecules that restrict the same functions in T cells. We foresee that recent technological advances may soon provide more efficient cancer treatments through precise definition and subsequent manipulation of transcriptional regulators underlying hypofunction of TIL.
